# Overexpression of Limb Bud and Heart Alleviates Sepsis-Induced Acute Lung Injury via Inhibiting the NLRP3 Inflammasome

**DOI:** 10.1155/2021/4084371

**Published:** 2021-01-23

**Authors:** Yifan Wang, Yang Shi, Xiaohong Zhang, Jing Fu, Feng Chen

**Affiliations:** Department of Emergency, Sichuan Academy of Medical Sciences & Sichuan Provincial People's Hospital, No. 32, The Second West Section of the First Ring Road, Qingyang District, Chengdu City, Sichuan Province 610000, China

## Abstract

**Objective:**

Sepsis is a leading cause of acute lung injury (ALI). This study attempted to investigate the effects of limb bud and heart (LBH) on the development of sepsis-induced ALI and its underlying mechanism of action.

**Methods:**

The sepsis-induced ALI mouse model was established by cecal ligation and puncture (CLP). The lung injury score and lung wet/dry weight (W/D) ratio were used to evaluate the lung injury. *In vitro*, ALI was simulated by lipopolysaccharide (LPS) treatment in A549 cells. The mRNA expression of LBH, NLRP3, ASC, and proinflammatory cytokines was measured by qRT-PCR. The viability of LPS-induced A549 cells was analyzed by MTT assay. Furthermore, western blot was performed to detect the protein expression of LBH, NLRP3, and ASC. LPS-induced A549 cells were treated with MCC950 (NLRP3 inflammasome inhibitor) to confirm the effect of LBH on NLRP3 inflammasome.

**Results:**

The mRNA and protein expression of LBH was decreased in sepsis-induced ALI. LBH overexpression reduced the lung injury score, lung W/D ratio, expression of proinflammatory cytokines, and NLRP3 inflammasome activation in sepsis-induced ALI mouse model. Additionally, LBH upregulation increased the viability, while it decreased the proinflammatory cytokine expression and NLRP3 inflammasome activation of LPS-induced A549 cells. Moreover, MCC950 reversed the promoting effects of LBH silencing on proinflammatory cytokine expression and NLRP3 inflammasome activation in LPS-induced A549 cells.

**Conclusions:**

LBH alleviated lung injury in sepsis-induced ALI mouse model by inhibiting inflammation and NLRP3 inflammasome, and restrained the inflammation by inhibiting NLRP3 inflammasome in LPS-induced A549 cells, providing a novel therapeutic target for ALI.

## 1. Introduction

Sepsis leads to multiple organ dysfunctions that can produce diverse critical illness [1]. Lung dysfunction, referred to as acute lung injury (ALI), is generally associated with sepsis [2]. ALI is characterized by pulmonary alveoli edema, mitochondrial dysfunction, and exuberant proinflammatory responses [3]. More than 40% of ALI patients have disseminated intravascular coagulation or hematological, cardiovascular, and neurological dysfunctions; all of which were related to higher mortality [4]. The anti-inflammatory drugs for ALI are inefficient, and the treatments of ALI require high costs [5, 6]. Therefore, it is essential to explore new approaches for the treatment of ALI.

Limb bud and heart (LBH) belongs to the intrinsically disordered protein family and plays pivotal role in human disease [7]. It has been documented that LBH participates in the development of inflammatory diseases. Matsuda et al. have reported that LBH inhibition enhances DNA damage, alters cell cycle kinetics, and promotes synovial inflammation in rheumatoid arthritis [8]. Chang et al. have displayed that LBH expression is low-expressed in systemic lupus erythematosus (SLE) patients and is associated with the pathogenesis of SLE [9]. LBH also has been proved to modulate the process of lung diseases. For instance, LBH downregulation in idiopathic pulmonary fibrosis patients is correlated with worse transplant-free survival and low forced vital capacity [10]. LBH predicts survival outcome and attenuates invasion and growth of human lung adenocarcinoma cells [11]. However, the function and underlying mechanism of LBH in the regulation of ALI is still unknown.

The NLRP3 inflammasome is a critical signaling node that accelerates the maturation of proinflammatory cytokines IL-18 and IL-1*β* [12]. It contains NLRP3, ASC, caspase-1, and NOD-like receptor [13]. NLRP3 inflammasome has been linked with various human inflammatory diseases including chronic kidney disease [14], gout [15], and ALI [16]. Increasing evidences have exhibited that numerous genes take part in the pathogenesis of ALI via regulating NLRP3 inflammasome. For instance, miR-223 declines the lipopolysaccharide- (LPS-) induced inflammation in ALI by suppressing NLRP3 inflammasome [17]. Apelin-13 administration protects against LPS-induced ALI via repressing NLRP3 inflammasome [18]. CORM-2 attenuates the NLRP3 inflammasome pathway, which restrains the inflammatory responses and related tissue injury in ALI [19]. Despite these reports, the specific regulatory relationship between LBH and NLRP3 inflammasome in ALI remains undefined.

In this study, we evaluated LBH expression in sepsis-induced ALI mouse model and explored the regulatory effects of LBH on lung injury, inflammation, and NLRP3 inflammasome activation. Additionally, we explored the function of LBH in regulating NLRP3 inflammasome activation and inflammation in LPS-induced A549 cells. Our results may provide a hopeful therapeutic target for ALI (Supplemental Figure 1).

## 2. Methods

### 2.1. Cecal Ligation and Puncture (CLP) Model of Sepsis

A total of 20 male C57BL/6 mice (6-8 weeks old) were purchased from Beijing Vital River Laboratory Animal Technology Co., Ltd. After one week of adjustment, sepsis-induced ALI mouse model was established. Simply, mice were anesthetized by intraperitoneal injection of sodium pentobarbital (35 mg/kg) and underwent CLP. The cecum was exposed and ligated 0.5 cm from the tip with 4-0 silk suture. A 22-gauge needle was used for one puncture site where a little bit of fecal contents were extruded. At last, the wounds were closed using a 4-0 silk suture. Similar procedures were performed on the mice of the sham group (*n* = 5), except ligation and puncture. All mice were resuscitated by subcutaneous injection of 1 ml of prewarmed saline. The animal experiments were permitted by the Ethics Committee of our hospital.

### 2.2. Injection of Lentivirus into Sepsis-Induced ALI Mouse Model

Recombinant lentiviruses carrying sequences encoding LBH (lv-LBH) and a negative control sequence (lv-NC) were constructed by Shanghai GenePharma (Shanghai, China). After lentivirus infection of the A549 cell, LBH-overexpressing cells (lv-LBH) and control cells (lv-NC) were gained through two-week puromycin (2 *μ*g/ml) screening. The sepsis-induced ALI mouse model was randomly divided into three groups (*n* = 5): the CLP group (the sepsis-induced ALI mouse model without treatment), the CLP+lv-LBH group (A549 cells infected with lv-LBH were instilled intranasally into the sepsis-induced ALI mouse model), and the CLP+lv-NC group (A549 cells infected with lv-NC were instilled intranasally into the sepsis-induced ALI mouse model). Mice were anesthetized with 50 mg/kg pentobarbital sodium and sacrificed by cervical dislocation. The left lung was collected for the measurement of the lung wet/dry weight (W/D) ratio assay. The right lung was stored at -80°C for the future experiments.

### 2.3. The Lung W/D Ratio

Then, the left lung was rinsed carefully, blotted, and weighed (wet weight). Subsequently, the left lung was dried in an oven at 60°C for 72 h and weighed (dry weight). The lung W/D ratio was calculated as (wet weight/dry weight) × 100%.

### 2.4. Hematoxylin-Eosin (HE) Staining

Lung tissues were fixed in 4% paraformaldehyde for 24 h, embedded in paraffin, cut into sections at 4*μ*mthickness, and stained with HE staining. By means of light microscopy (Nikon, Eclipse 80i, Tokyo, Japan), the degree of histological injury in lung tissues was observed.

### 2.5. Cell Culture and Treatment

Human respiratory epithelial A549 cells were obtained from American Type Culture Collection (Manassas, VA, USA). A549 cells were cultured in RPMI-1640 (Gibco, Erie, NY, USA) with 10% fetal bovine serum (FBS, Invitrogen, Carlsbad, NY, USA) at 37°C containing 5% CO_2_. A549 cells treated with 100 ng/ml LPS for 24 h were regarded as the sepsis-induced ALI model at the cellular level (LPS-induced A549 cells) [20–22].

### 2.6. Cell Transfection

The short hairpin- (sh-) LBH, sh-NC, pcDNA3.1-NC (pcDNA3.1), and pcDNA3.1-LBH (oe-LBH) were synthesized by GenePharma (Shanghai, China). LPS-inducedA549 cells grown to 85% confluence were transfected with these above agents using Lipofectamine 3000 (Invitrogen). The LPS-induced A549 cells in the blank or NC group did not receive any transfection. After the transfection, LPS-induced A549 cells or LPS-induced A549 cells transfected with sh-LBH were treated with 10 *μ*M MCC950 (NLRP3 inflammasome inhibitor) for another 48 h.

### 2.7. Western Blot

Total proteins were extracted from tissues and cells, and then transferred into SDS-PAGE. Separated protein was transferred onto polyvinylidene fluoride membranes, blocked with 5% skimmed milk, and incubated at 4°C overnight with primary antibodies, including anti-LBH (1 : 1000, SAB1304367MSDS, Sigma), anti-NLRP3 (1 : 1000, ABF23MSDS, Sigma), anti-ASC (1 : 1000, A1601MSDS, Sigma), and anti-*β*-actin (1 : 5000, SAB2701711MSDS, Sigma). Afterwards, the membranes were subjected to HRP-labeled goat anti-rabbit IgG (1 : 5000, 12-348MSDS, Sigma) secondary antibody at 25°C for 1 h. The immunoblots were measured through ECL system and quantified by Image Lab software (Bio-Rad, Hercules, CA, USA).

### 2.8. Quantitative Real-Time Polymerase Chain Reaction (qRT-PCR)

The mRNA expression of specific genes (LBH, IL-1*β*, IL-6, IL-18, NLRP3, and ASC) was measured by qRT-PCR as previously described [23–25]. Total RNA was extracted from tissues and cells using the TRIzol reagent (Invitrogen). Then, cDNA samples were attained through reverse transcription using PrimeScript RT Reagent Kit (Takara, Japan). Next, qRT-PCR was conducted on 7500 Real-Time PCR System (Applied Biosystems, Waltham, MA, USA). The reaction conditions were as follows: 95°C for 10 min, followed by 40 cycles at 95°C for 10 s, 60°C for 20 s, and 72°C for 34 s. GAPDH was used as internal control for LBH, and *β*-actin was used for others. Relative expression level was calculated by the 2^-*ΔΔ*Ct^ method (ΔΔCt = ΔCt1 − ΔCt2; ΔCt1 = Ct value of target gene in the test group − Ct value of internal control in the test group; ΔCt2 = Ct value of target gene in the control group − Ct value of internal control in the control group). The primer sequences are shown in Table 1.

### 2.9. MTT Assay

The LPS-induced A549 cells were seeded into 96-well plates (2 × 10^3^ cells/well) and cultured with 5% CO_2_ at 37°C for 72 h. Cell viability was measured using the MTT cell proliferation assay kit (Sigma) according to the guidelines.

### 2.10. Statistical Analysis

Data statistical analysis was performed using GraphPad Prism 7.0 (GraphPad, San Diego, CA, USA). Data were presented as the mean ± standard deviation. The differences between two groups or among multiple groups were assessed by Student's *t*-test or one-way ANOVA followed by Tukey's post hoc test. The correlation significance was evaluated by Pearson correlation analysis. Differences were considered statistically significant at *P* < 0.05.

## 3. Results

### 3.1. LBH Expression Was Decreased in the Lung Tissues of Sepsis-Induced ALI

**Figure 1:**
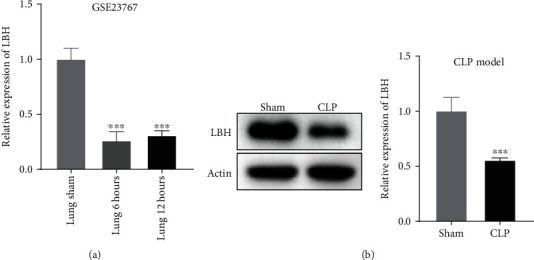
LBH expression was decreased in the lung tissues of sepsis-induced acute lung injury (ALI). (a) The Gene Expression Omnibus (GEO, accession number GSE23767) was used to analyze the differential expression of LBH in sepsis-induced ALI model. ^∗∗∗^*P* < 0.001 vs. lung sham. (b) The protein expression of LBH in lung tissues was detected by western blot. ^∗∗∗^*P* < 0.001 vs. sham.

The Gene Expression Omnibus (GEO, accession number GSE23767) was used to analyze the differential expression of LBH in sepsis-induced ALI model. At 6 and12 h after CLP operation, the LBH expression in lung tissues from CLP mice was considerably downregulated compared with sham-operated mice (*P* < 0.001, Figure 1(a)). Furthermore, western blot uncovered that the LBH protein expression in lung tissues was dramatically inhibited in sepsis-induced ALI mouse model (*P* < 0.001, Figure 1(b)).

### 3.2. LBH Increased the Viability and Attenuated Inflammation of LPS-Induced A549 Cells

**Figure 2:**
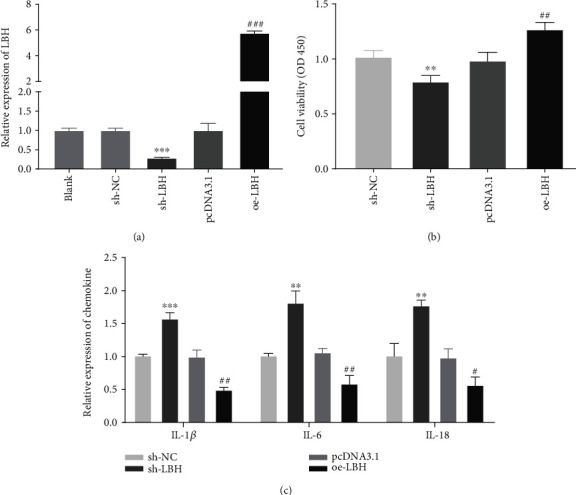
LBH increased the viability and attenuated inflammation of lipopolysaccharide- (LPS-) induced A549 cells. (a) The transfection efficiency of sh-NC, sh-LBH, pcDNA-NC (pcDNA3.1), and pcDNA3.1-LBH (oe-LBH) was demonstrated by using qRT-PCR. ^∗∗∗^*P* < 0.001 vs. sh-NC, ^###^*P* < 0.001 vs. pcDNA3.1. (b) The viability of LPS-induced A549 cells was measured by MTT assay. ^∗∗^*P* < 0.01 vs. sh-NC, ^##^*P* < 0.01 vs. pcDNA3.1. (c) qRT-PCR was performed to confirm the expression level of IL-1*β*, IL-6, and IL-18 in LPS-induced A549 cells. ^∗∗^*P* < 0.01 and ^∗∗∗^*P* < 0.001 vs. sh-NC, ^#^*P* < 0.05 and ^##^*P* < 0.01 vs. pcDNA3.1.

To explore the effect of LBH on ALI *in vitro*, we performed the LPS induction to produce LPS-induced ALI in A549 cells. qRT-PCR exhibited that the LBH expression was inhibited or enhanced by the transfection of sh-LBH oroe-LBH into LPS-induced A549 cells (*P* < 0.001, Figure 2(a)). MTT assay displayed that LBH inhibition markedly declined the viability of LPS-inducedA549 cells, while LBH overexpression obviously elevated the cell viability (*P* < 0.01, Figure 2(b)). As depicted in Figure 2(c), LBH knockdown visibly increased the expression of IL-1*β*, IL-6, and IL-18 in LPS-induced A549 cells (*P* < 0.01). In contrary, the expression of above proinflammatory cytokines was strikingly reduced by LBH overexpression (*P* < 0.05).

### 3.3. LBH Attenuated NLRP3 Inflammasome Activation in LPS-Induced A549 Cells

**Figure 3:**
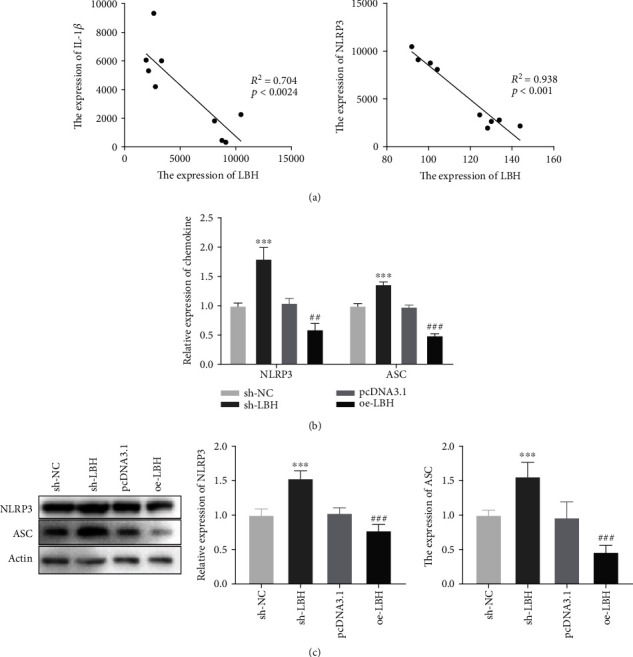
LBH attenuated NLRP3 inflammasome activation in LPS-induced A549 cells. (a) The Gene Expression Omnibus (GEO, accession number GSE23767) exhibited that the expression of LBH was inversely related to the expression of IL-1*β* and NLRP3 in acute lung injury (ALI). (b) The mRNA expression of NLRP3 and ASC in LPS-induced A549 cells was measured by qRT-PCR. ^∗∗∗^*P* < 0.001 vs. sh-NC, ^##^*P* < 0.01 and ^###^*P* < 0.001 vs. pcDNA3.1. (c) Western blot was performed to detect the protein expression of NLRP3 and ASC in LPS-induced A549 cells. ^∗∗∗^*P* < 0.001 vs. sh-NC, ^###^*P* < 0.001 vs. pcDNA3.1.

As illustrated in Figure 3(a), the GEO (accession number GSE23767) was used to analyze the correlation between LBH and IL-1*β* expression and LB Hand NLRP3 expression in sepsis-induced ALI model. The results displayed that the expression of LBH was inversely related to the expression of IL-1*β* (*R*^2^ = 0.704, *P* < 0.0024) and NLRP3 (*R*^2^ = 0.938, *P* < 0.001) in ALI. To investigate the effect of LBH on NLRP3 inflammasome activation, the mRNA and protein expression of NLRP3 and ASC was detected by qRT-PCR and western blot. The results displayed that LBH silencing obviously increased the NLRP3 and ASC mRNA expression (*P* < 0.001), while LBH overexpression markedly decreased the NLRP3 and ASC expression in LPS-induced A549 cells (*P* < 0.01, Figure 3(b)). Moreover, western blot assay revealed that downregulation or upregulation of LBH could dramatically elevate or reduce the protein expression of NLRP3and ASC in LPS-induced A549 cells, respectively (*P* < 0.001, Figure 3(c)).

### 3.4. LBH Reduced Inflammation through Inhibiting NLRP3 Inflammasome Activation in LPS-Induced A549 Cells

**Figure 4:**
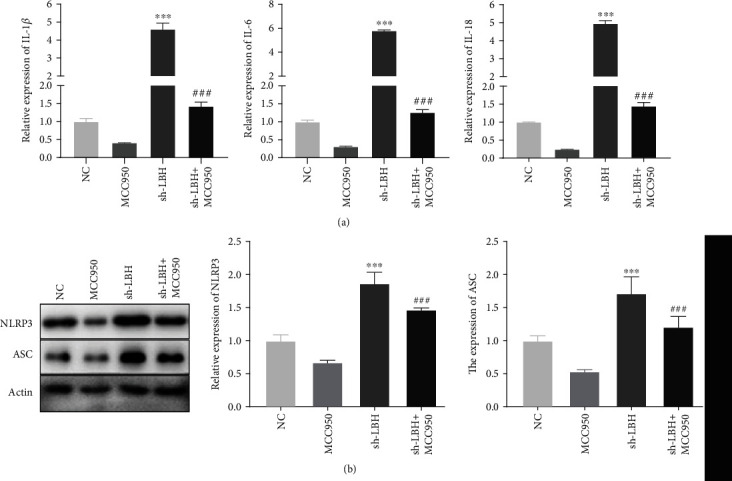
LBH reduced inflammation through inhibiting NLRP3 inflammasome activation in LPS-induced A549 cells. (a) MCC950 reversed the promoting effects of sh-LBH on the expression of IL-1*β*, IL-6, and IL-18 in LPS-induced A549 cells. ^∗∗∗^*P* < 0.001 vs. NC, ^###^*P* < 0.001 vs. sh-LBH. (b) MCC950 eliminated the facilitation effects on the NLRP3 and ASC protein expression in LPS-induced A549 cells caused by sh-LBH. ^∗∗∗^*P* < 0.001 vs. NC, ^###^*P* < 0.001 vs. sh-LBH.

NLRP3inflammatory activation was inhibited by the transfection of MCC950 (NLRP3 inflammasome inhibitor) in LPS-induced A549 cells, and then, the expression of inflammatory cytokines was examined by qRT-PCR. The results showed that LBH knockdown considerably increased the expression of IL-1*β*, IL-6, and IL-18 in LPS-induced A549 cells (*P* < 0.001), and MCC950 reversed the promoting effects of LBH knockdown on the expression of inflammatory cytokines in LPS-induced A549 cells (*P* < 0.001, Figure 4(a)). Moreover, sh-LBH could visibly enhance the NLRP3 and ASC protein expression in LPS-induced A549 cells (*P* < 0.001), and MCC950 eliminated the facilitation effects on the NLRP3 and ASC protein expression in LPS-induced A549 cells caused by LBH silencing (*P* < 0.001, Figure 4(b)).

### 3.5. LBH Overexpression Alleviated the Lung Injury of Sepsis-Induced ALI

**Figure 5:**
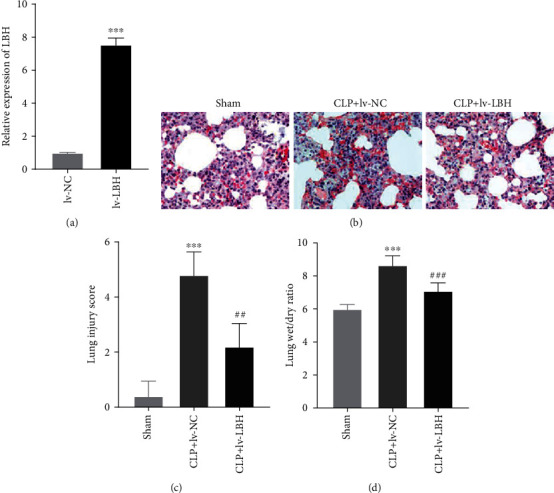
LBH overexpression alleviated the lung injury of sepsis-induced acute lung injury (ALI). (a) The LBH expression in A549 cells was measured by qRT-PCR. ^∗∗∗^*P* < 0.001 vs. lv-NC. (b) The histopathological change in lung tissues was observed by hematoxylin-eosin (HE) staining. (c) The degree of lung injury was evaluated by lung injury score. ^∗∗∗^*P* < 0.001 vs. sham, ^##^*P* < 0.01 vs. CLP+lv-NC. (d) The lung wet/dry weight (W/D) ratio was measured. ^∗∗∗^*P* < 0.001 vs. sham, ^###^*P* < 0.001 vs. CLP+lv-NC.

As shown in Figure 5(a), the LBH expression in A549 cells was increased by the infection of lv-LBH (*P* < 0.001). Then, we further explore the biological function of LBH in sepsis-induced ALI *in vivo*. The degree of injury in lung tissues was observed by HE staining. As exhibited in Figure 5(b), the lung morphology of mice in the CLP+lv-NC group changed greatly, including neutrophil infiltration, hemorrhage, interstitial edema, alveolar disarray, and thickness of the alveolar septum. However, these histological changes were ameliorated by infection oflv-LBH. Moreover, the lung injury score was considerably elevated in sepsis-induced ALI mouse model (*P* < 0.001, Figure 5(c)). LBH overexpression could markedly reduce the lung injury score in sepsis-induced ALI mouse model (*P* < 0.01, Figure 5(c)). Additionally, the lung W/D ratio was obviously increased in the CLP group comparing with the sham group (*P* < 0.001, Figure 5(d)). The lung W/D ratio in sepsis-induced ALI mouse model was visibly decreased by LBH overexpression (*P* < 0.001, Figure 5(d)).

### 3.6. LBH Overexpression Attenuated Inflammation and NLRP3 Inflammasome Activation in Sepsis-Induced ALI Mouse Model

**Figure 6:**
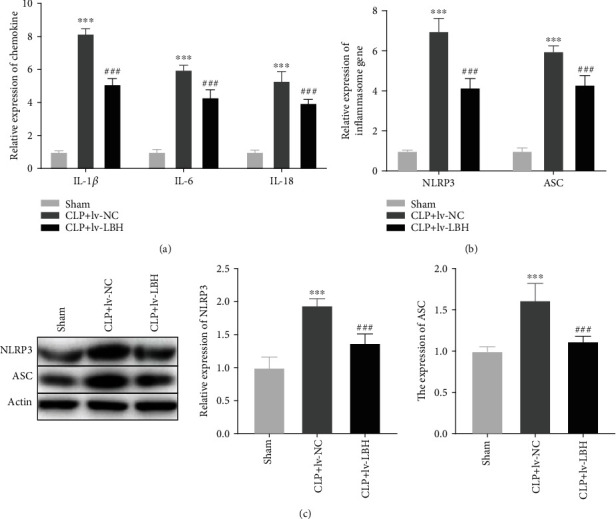
LBH overexpression attenuated inflammation and NLRP3 inflammasome activation in sepsis-induced acute lung injury (ALI) mouse model. (a) qRT-PCR was performed to confirm the expression of IL-1*β*, IL-6, and IL-18 in lung tissues. ^∗∗∗^*P* < 0.001 vs. sham, ^###^*P* < 0.001 vs. CLP+lv-NC. (b, c) The mRNA and protein expression of NLRP3 and ASC in lung tissues was detected by qRT-PCR and western blot, respectively. ^∗∗∗^*P* < 0.001 vs. sham, ^###^*P* < 0.001 vs. CLP+lv-NC.

As depicted in Figure 6(a), IL-1*β*, IL-6, and IL-18 expression in the CLP+lv-NC was higher than that in the sham group (*P* < 0.001). The expression of IL-1*β*, IL-6, and IL-18 in lung tissues of sepsis-induced ALI mouse model was obviously decreased by the infection of lv-LBH (*P* < 0.001). Besides, the expression of NLRP3 and ASC in the CLP+lv-NC group was visibly increased compared to the sham group (*P* < 0.001, Figure 6(b)). LBH overexpression could markedly downregulate the expression of NLRP3 and ASC in lung tissues of sepsis-induced ALI mouse model (*P* < 0.001, Figure 6(b)). Moreover, western blot assay discovered that the protein expression of NLRP3 and ASC in the lv-NC group was considerably enhanced compared to those in the sham group (*P* < 0.001, Figure 6(c)). Upregulation of LBH could significantly reduce the NLRP3 and ASC protein expression in lung tissues of sepsis-induced ALI mouse model (*P* < 0.001, Figure 6(c)).

## 4. Discussion

The sepsis-induced ALI mouse model is frequently recognized as a model of human ALI [26, 27]. LBH is regarded as a pivotal regulator in embryonic development and was downregulated in several diseases such as rheumatoid arthritis [28], SLE [9], and nasopharyngeal carcinoma [29]. In this study, LBH protein expression was decreased in the lung tissues of sepsis-induced ALI mouse model. Hence, LPS-induced A549 cells were selected and treated with LBH as to further confirm the biochemical mechanisms underlying the effect of LBH in pathogenesis of sepsis-induced ALI. The present study provided *in vivo* and *vitro* evidences that LBH plays critical role in the development of sepsis-induced ALI.

It has been proved that some genes have protective effect against ALI by increasing lung cell viability. For instances, Nrf2 protects against ALI by elevating the lung cell viability [30]. Upregulation of 4-PBA significantly increases the viability of LPS-induced A549 cells [31]. Here, LBH overexpression increased the viability of LPS-induced A549 cells, suggesting that LBH may alleviate ALI through promoting cell viability. Additionally, previous studies have demonstrated that inhibition of inflammatory responses attenuates ALI progression. For instances, Miz1 inhibits the contents of IL-1*β* and IL-6 in LPS-induced lung cells [32]. Elevation of lncRNA TUG1 reduces LPS-induced inflammation in pulmonary microvascular endothelial cells [33]. In the present study, LBH treatment declined the expression of IL-1*β*, IL-6, and IL-18 in the LPS-induced A549 cells, indicating that LBH may ameliorate ALI via inhibiting inflammation. Altogether, these data indicate that LBH overexpression may protect against ALI by increasing cell viability and attenuating inflammation *in vitro*.

NLRP3 inflammasome is responsible for proinflammatory cytokine maturation and secretion, and can be activated in response to cellular stresses during lung injury [34]. Deletion of NLRP3 inflammasome decreases lung epithelial cell death and attenuates the recruitment of inflammatory cells and the elevation of proinflammatory cytokines, exerting a protective effect against ALI [35]. In this study, analyzing the database from the GEO, the results exhibited that the LBH expression was negatively associated with the NLRP3 expression. The abovementioned data indicated that LBH may protect against ALI through negatively regulating NLRP3. The formation of mature active form of IL-1*β* is highly dependent on the activation of NLRP3 inflammasomes during the process of ALI [36–38]. IL-1*β* induces vascular endothelial and alveolar epithelial permeability, leading to alveolar edema in ALI [39]. Here, the database of GEO displayed that the LBH expression was negatively related to the IL-1*β* expression, suggesting that LBH may attenuate ALI by negatively regulating IL-1*β*.

NLRP3 and ASC are the important components of NLRP3 inflammasome [40]. The level of NLRP3 protein expression has been shown to be a limiting step in controlling inflammasome activation [41]. Upon activation, NLRP3 forms an inflammasome with the adapter ASC [42]. In this study, LBH overexpression decreased the mRNA and protein expression of NLRP3 and ASC in LPS-induced A549 cells, indicating that LBH may inhibit the NLRP3 inflammasome activation in LPS-induced A549 cells. Previous researches have confirmed that inhibition of the NLRP3 inflammasome activation can suppress the development of ALI. For instances, hemin alleviates sepsis-induced ALI by inhibiting activation of NLRP3 inflammasome and attenuating inflammatory response *in vitro* [43]. Morin exerts a protective effect on ALI and attenuates proinflammatory cytokine secretion via reducing activation of NLRP3 inflammasome [44]. Dihydromyricetin alleviates inflammatory reactions by suppressing the CLP-induced NLRP3 inflammasome pathway in sepsis-induced ALI [45]. Here, LBH overexpression inhibited the expression of IL-1*β*, IL-6, and IL-18 in LPS-induced A549 cells. Given that LBH inhibited the NLRP3 inflammasome activation in LPS-induced A549 cells, we speculate that LBH may decrease the expression of IL-1*β*, IL-6, and IL-18 through inhibiting NLRP3 inflammasome activation. To further verify the speculation, we treated LPS-induced A549 cells with MCC950, an NLRP3 inflammasome inhibitor. Encouragingly, our results showed that MCC950 effectively reversed the promoting effects of LBH silencing on the expression of IL-1*β*, IL-6, and IL-18 in the LPS-induced A549 cells. Taken together, we suggest that LBH may alleviate the inflammation in LPS-induced A549 cells via attenuating NLRP3 inflammasome activation.

To further explore the effects of LBH on lung injury, inflammatory responses, and NLRP3 inflammasome activation in sepsis-induced ALI, we performed *in vivo* experiments in sepsis-induced ALI mouse model. The lung histological changes, lung injury score, and lung W/D ratio are the important indexes to evaluate the degree of lung injury in the development of ALI [46]. The therapeutic effects of many drugs on ALI are reflected in the reduction of these indicators. For instances, maresin 1 alleviates the histological changes of the lung and reduces the lung W/D ratio in LPS-induced ALI in mice [47]. Melatonin treatment declines the lung injury score in LPS-induced ALI mouse model [48]. Ketamine treatment prevents CLP-induced alveolar wall thickening and accumulation of neutrophils, and decreases the lung W/D ratio in sepsis-induced ALI [49]. In this study, overexpression of LBH reduced the lung histological changes, lung injury score, and lung W/D ratio in sepsis-induced ALI mouse model, indicating that LBH may act as a protective gene for treating sepsis-induced ALI *in vivo*. Inflammation has been identified as the major cause that leads to lung injury [50]. NLRP3 inflammasome has been shown to be activated in ALI models [51]. In this study, LBH overexpression decreased the expression of proinflammatory cytokines and NLRP3 inflammasome components in lung tissues of sepsis-induced ALI mouse model, indicating that LBH treatment attenuated the inflammation and NLRP3inflammasome activation in sepsis-induced ALI mice. To sum up, we indicated that LBH overexpression may alleviate the progression of sepsis-induced ALI *in vivo* by inhibiting the lung injury, inflammatory responses, and NLRP3inflammasome activation.

## 5. Conclusion

In summary, the protein expression of LBH was decreased in the lung tissues of sepsis-induced ALI mouse model. *In vitro*, overexpression of LBH reduced the viability and expression of proinflammatory cytokines (IL-1*β*, IL-6, and IL-18) in LPS-induced A549 cells, suggesting that overexpression of LBH may protect against ALI by increasing cell viability and attenuating inflammation *in vitro*. Upregulation of LBH inhibited the mRNA and protein expression of NLRP3 and ASC in LPS-induced A549 cells. The mechanical experiment demonstrated that MCC950 (NLRP3 inflammasome inhibitor) effectively reversed the promoting effects of LBH silencing on the expression of IL-1*β*, IL-6, and IL-18 in the LPS-induced A549 cells, indicating that upregulation of LBH may alleviate the inflammation via attenuating NLRP3 inflammasome activation in LPS-induced A549 cells. *In vivo*, overexpression of LBH decreased the lung histological changes, lung injury score, lung W/D ratio, expression of IL-1*β*, IL-6, and IL-18, and activation of NLRP3inflammasome in lung tissues of sepsis-induced ALI mouse model. Therefore, LBH may be a promising therapeutic target for ALI. However, there were several limitations to our study. First, there are many other downstream signaling pathways of LBH that have not yet been determined in sepsis-induced ALI. Second, due to the lack of clinical samples with follow-up information, it was difficult to determine the clinical significance of LBH. Third, the regulatory relationship between LBH and NLRP3 inflammasome on inflammatory responses in sepsis-induced ALI is limited to the cellular level and *in vivo* experiments are needed. Related experiments will be considered in our future studies (Supplemental Figure 2).

## Figures and Tables

**Table 1:** Primer sequences used in quantitative real-time polymerase chain reaction (qRT-PCR).

Name of primer	Sequences (5′-3′)
LBH-F	CCATTCACTGCCCCGACTAT
LBH-R	TTTCGCTGTCTCTTCGCAGT
GAPDH-F	GAAGGTGAAGGTCGGAGTCA
GAPDH-R	GACAAGCTTCCCGTTCTCAG
NLRP3-F	TGGGTTCTGGTCAGACACGAG
NLRP3-R	GGCGGGTAATCTTCCAAATGC
ASC-F	GGAGTCGTATGGCTTGGAGC
ASC-R	CGTCCACTTCTGTGACCCTG
IL-1*β*-F	GCCCTAAACAGATGAAGTGCTC
IL-1*β*-R	GAACCAGCATCTTCCTCAG
IL-6-F	GGAAATCGTGGAAATGAG
IL-6-R	AGGACTCTGGCTTTGTCT
IL-18-F	ACAGGCCTGACATCTTCTGC
IL-18-R	CCTTGAAGTTGACGCAAGAGT
*β*-Actin-F	CCCGCGAGTACAACCTTCTT
*β*-Actin-R	TCATCCATGGCGAACTGGTG

## Data Availability

All data generated or analyzed during this study are included in this published article.

## Supplementary Materials

Supplementary materials Supplemental Figure 1: a flowchart of research methodology. Supplemental Figure 2: a structure chart of regulatory effect of LBH on the progression of sepsis-induced acute lung injury (ALI) in this study.

## References

[1] Gotts J. E., Matthay M. A. (2016). Sepsis: pathophysiology and clinical management. *BMJ*.

[2] Fujishima S. (2016). Organ dysfunction as a new standard for defining sepsis. *Inflammation and Regeneration*.

[3] Wheeler A. P., Bernard G. R. (2007). Acute lung injury and the acute respiratory distress syndrome: a clinical review. *Lancet*.

[4] Fujishima S., Gando S., Daizoh S. (2016). Infection site is predictive of outcome in acute lung injury associated with severe sepsis and septic shock. *Respirology*.

[5] Yamashita C. M., Lewis J. F. (2012). Emerging therapies for treatment of acute lung injury and acute respiratory distress syndrome. *Expert Opinion on Emerging Drugs*.

[6] Standiford T. J., Ward P. A. (2016). Therapeutic targeting of acute lung injury and acute respiratory distress syndrome. *Translational Research*.

[7] Al-Ali H., Rieger M. E., Seldeen K. L., Harris T. K., Farooq A., Briegel K. J. (2010). Biophysical characterization reveals structural disorder in the developmental transcriptional regulator LBH. *Biochemical and Biophysical Research Communications*.

[8] Matsuda S., Hammaker D., Topolewski K. (2017). Regulation of the cell cycle and inflammatory arthritis by the transcription cofactor LBH gene. *Journal of Immunology*.

[9] Chang Y., Sheng Y., Cheng Y. (2016). Downregulated expression of LBH mRNA in peripheral blood mononuclear cells from patients with systemic lupus erythematosus. *The Journal of Dermatology*.

[10] Herazo-Maya J., Noth I., Juan-Guardela B. M. (2015). Limb bud and heart, a novel developmental gene is a biomarker of idiopathic pulmonary fibrosis severity, progression and outcome. *American Journal of Respiratory and Critical Care Medicine*.

[11] Deng M., Yu R., Wang S. (2018). Limb-bud and heart attenuates growth and invasion of human lung adenocarcinoma cells and predicts survival outcome. *Cellular Physiology and Biochemistry*.

[12] He Y., Hara H., Nunez G. (2016). Mechanism and regulation of NLRP3 inflammasome activation. *Trends in Biochemical Sciences*.

[13] Jo E. K., Kim J. K., Shin D. M., Sasakawa C. (2016). Molecular mechanisms regulating NLRP3 inflammasome activation. *Cellular & Molecular Immunology*.

[14] Vilaysane A., Chun J., Seamone M. E. (2010). The NLRP3 inflammasome promotes renal inflammation and contributes to CKD. *Journal of the American Society of Nephrology*.

[15] Kingsbury S. R., Conaghan P. G., McDermott M. F. (2011). The role of the NLRP3 inflammasome in gout. *Journal of Inflammation Research*.

[16] Grailer J. J., Canning B. A., Kalbitz M. (2014). Critical role for the NLRP3 inflammasome during acute lung injury. *Journal of Immunology*.

[17] Yan Y., Lu K., Ye T., Zhang Z. (2019). MicroRNA-223 attenuates LPS-induced inflammation in an acute lung injury model via the NLRP3 inflammasome and TLR4/NF-*κ*B signaling pathway via RHOB. *International Journal of Molecular Medicine*.

[18] Zhang H., Chen S., Zeng M. (2018). Apelin-13 administration protects against LPS-induced acute lung injury by inhibiting NF-*κ*B pathway and NLRP3 inflammasome activation. *Cellular Physiology and Biochemistry*.

[19] Jiang L., Fei D., Gong R. (2016). CORM-2 inhibits TXNIP/NLRP3 inflammasome pathway in LPS-induced acute lung injury. *Inflammation Research*.

[20] Li P., Yao Y., Ma Y., Chen Y. (2019). miR-150 attenuates LPS-induced acute lung injury via targeting AKT3. *International Immunopharmacology*.

[21] Luo X., Liu R., Zhang Z., Chen Z., He J., Liu Y. (2019). Mitochondrial division inhibitor 1 attenuates mitophagy in a rat model of acute lung injury. *BioMed Research International*.

[22] Shao L., Meng D., Yang F., Song H., Tang D. (2017). Irisin-mediated protective effect on LPS-induced acute lung injury via suppressing inflammation and apoptosis of alveolar epithelial cells. *Biochemical and Biophysical Research Communications*.

[23] Siddiqui A. J., Bhardwaj J., Goyal M. (2015). Assessment of real-time method to detect liver parasite burden under different experimental conditions in mice infected with Plasmodium yoelii sporozoites. *Microbial Pathogenesis*.

[24] Siddiqui A. J., Bhardwaj J., Puri S. K. (2012). mRNA expression of cytokines and its impact on outcomes after infection with lethal and nonlethal Plasmodium vinckei parasites. *Parasitology Research*.

[25] Livak K. J., Schmittgen T. D. (2001). Analysis of relative gene expression data using real-time quantitative PCR and the 2^−*ΔΔ*CT^ method. *Methods*.

[26] Kitzmiller L., Ledford J. R., Hake P. W., O'Connor M., Piraino G., Zingarelli B. (2019). Activation of AMP-activated protein kinase by A769662 ameliorates sepsis-induced acute lung injury in adult mice. *Shock*.

[27] Aziz M., Ode Y., Zhou M. (2018). B-1a cells protect mice from sepsis-induced acute lung injury. *Molecular Medicine*.

[28] Ekwall A. K., Whitaker J. W., Hammaker D., Bugbee W. D., Wang W., Firestein G. S. (2015). The rheumatoid arthritis risk gene LBH regulates growth in fibroblast-like synoviocytes. *Arthritis & Rhematology*.

[29] Liu Q., Guan X., Lv J., Li X., Wang Y., Li L. (2015). Limb-bud and heart (LBH) functions as a tumor suppressor of nasopharyngeal carcinoma by inducing G1/S cell cycle arrest. *Scientific Reports*.

[30] Yan J., Li J., Zhang L. (2018). Nrf2 protects against acute lung injury and inflammation by modulating TLR4 and Akt signaling. *Free Radical Biology and Medicine*.

[31] Zeng M., Sang W., Chen S. (2017). 4-PBA inhibits LPS-induced inflammation through regulating ER stress and autophagy in acute lung injury models. *Toxicology Letters*.

[32] Do-Umehara H. C., Chen C., Urich D. (2013). Suppression of inflammation and acute lung injury by Miz1 via repression of C/EBP-*δ*. *Nature Immunology*.

[33] Qiu N., Xu X., He Y. (2020). lncRNA TUG1 alleviates sepsis-induced acute lung injury by targeting miR-34b-5p/GAB1. *BMC Pulmonary Medicine*.

[34] Lee S., Suh G. Y., Ryter S. W., Choi A. M. (2016). Regulation and function of the nucleotide binding domain leucine-rich repeat-containing receptor, pyrin domain-containing-3 inflammasome in lung disease. *American Journal of Respiratory Cell and Molecular Biology*.

[35] Fukumoto J., Fukumoto I., Parthasarathy P. T. (2013). NLRP3 deletion protects from hyperoxia-induced acute lung injury. *American Journal of Physiology. Cell Physiology*.

[36] Jones H. D., Crother T. R., Gonzalez-Villalobos R. A. (2014). The NLRP3 inflammasome is required for the development of hypoxemia in LPS/mechanical ventilation acute lung injury. *American Journal of Respiratory Cell and Molecular Biology*.

[37] Garlanda C., Dinarello C. A., Mantovani A. (2013). The interleukin-1 family: back to the future. *Immunity*.

[38] Elliott E. I., Sutterwala F. S. (2015). Initiation and perpetuation of NLRP3 inflammasome activation and assembly. *Immunological Reviews*.

[39] Ganter M. T., Roux J., Miyazawa B. (2008). Interleukin-1beta causes acute lung injury via alphavbeta5 and alphavbeta6 integrin-dependent mechanisms. *Circulation Research*.

[40] Sutterwala F. S., Haasken S., Cassel S. L. (2014). Mechanism of NLRP3 inflammasome activation. *Annals of the New York Academy of Sciences*.

[41] Bauernfeind F. G., Horvath G., Stutz A. (2009). Cutting edge: NF-kappaB activating pattern recognition and cytokine receptors license NLRP3 inflammasome activation by regulating NLRP3 expression. *Journal of Immunology*.

[42] Stutz A., Kolbe C. C., Stahl R. (2017). NLRP3 inflammasome assembly is regulated by phosphorylation of the pyrin domain. *The Journal of Experimental Medicine*.

[43] Luo Y. P., Jiang L., Kang K. (2014). Hemin inhibits NLRP3 inflammasome activation in sepsis-induced acute lung injury, involving heme oxygenase-1. *International Immunopharmacology*.

[44] Tianzhu Z., Shihai Y., Juan D. (2014). The effects of morin on lipopolysaccharide-induced acute lung injury by suppressing the lung NLRP3 inflammasome. *Inflammation*.

[45] Wang Y. C., Liu Q. X., Zheng Q. (2019). Dihydromyricetin alleviates sepsis-induced acute lung injury through inhibiting NLRP3 inflammasome-dependent pyroptosis in mice model. *Inflammation*.

[46] Butt Y., Kurdowska A., Allen T. C. (2016). Acute lung injury: a clinical and molecular review. *Archives of Pathology & Laboratory Medicine*.

[47] Gong J., Wu Z. Y., Qi H. (2014). Maresin 1 mitigates LPS-induced acute lung injury in mice. *British Journal of Pharmacology*.

[48] Zhang Y., Li X., Grailer J. J. (2016). Melatonin alleviates acute lung injury through inhibiting the NLRP3 inflammasome. *Journal of Pineal Research*.

[49] Li K., Yang J., Han X. (2016). Ketamine attenuates sepsis-induced acute lung injury via regulation of HMGB1-RAGE pathways. *International Immunopharmacology*.

[50] Mokra D., Kosutova P. (2015). Biomarkers in acute lung injury. *Respiratory Physiology & Neurobiology*.

[51] Hosseinian N., Cho Y., Lockey R. F., Kolliputi N. (2015). The role of the NLRP3 inflammasome in pulmonary diseases. *Therapeutic Advances in Respiratory Disease*.

