# Supplementary material for: Overexpression of Limb Bud and Heart Alleviates Sepsis-Induced Acute Lung Injury via Inhibiting the NLRP3 Inflammasome
Source: Biomed Res Int. 2021 Jan 23;2021:4084371. doi: 10.1155/2021/4084371 (PMC7847343; doi:10.1155/2021/4084371)

Supplemental Figure 1 A flowchart of research methodology.


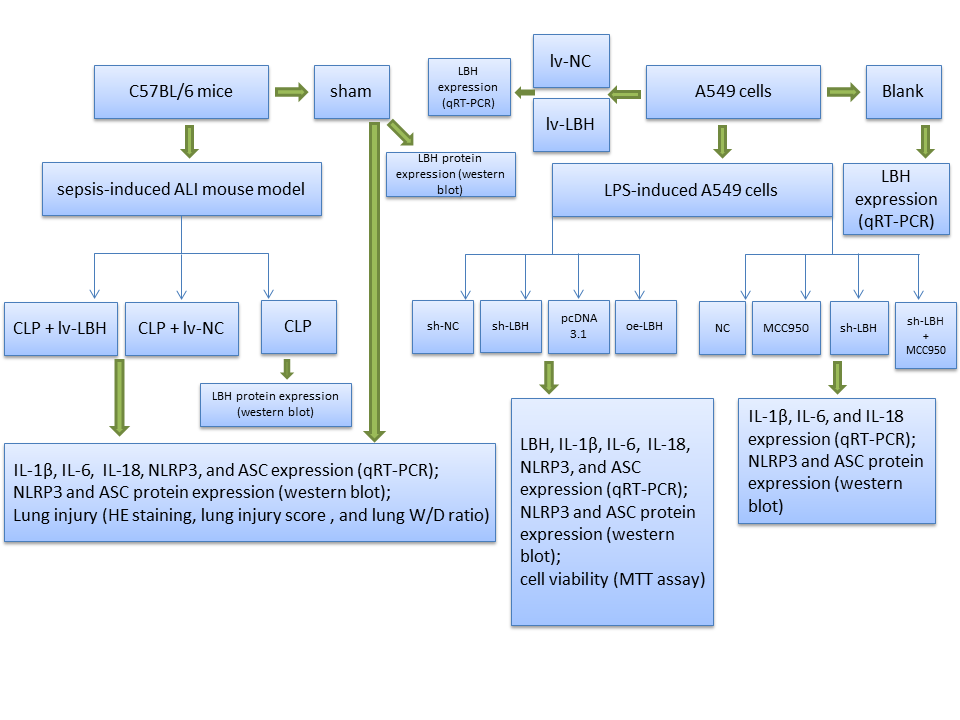


Supplemental Figure 2 A structure chart of regulatory effect of LBH on the progression of sepsis-induced acute lung injury (ALI) in this study.


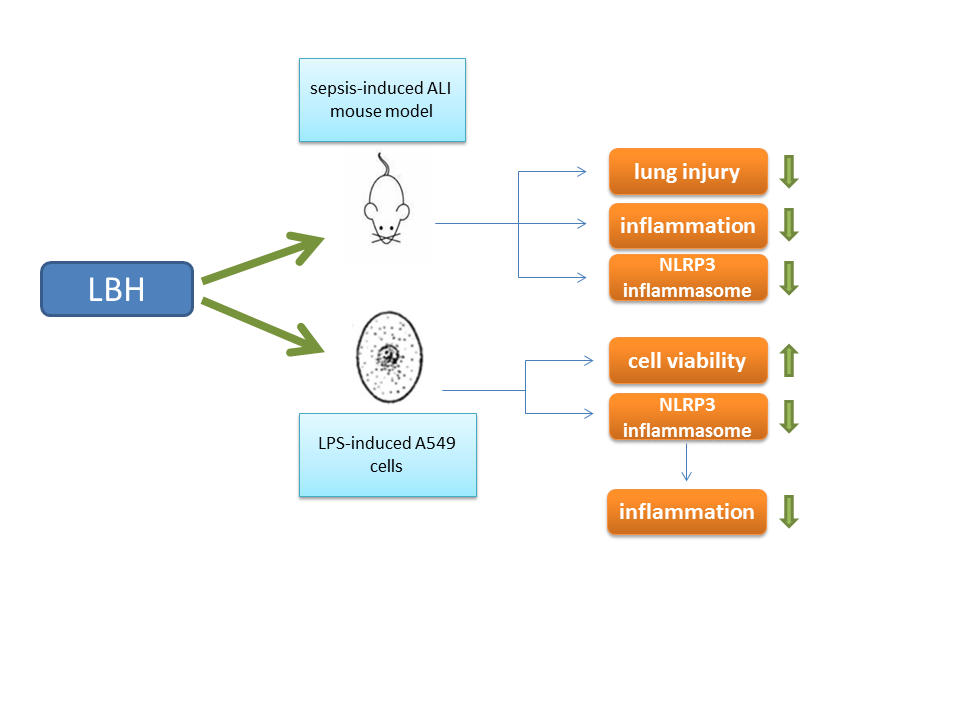

Supplement: Supplementary materials — Supplemental Figure 1: a flowchart of research methodology. Supplemental Figure 2: a structure chart of regulatory effect of LBH on the progression of sepsis-induced acute lung injury (ALI) in this study. [file 4084371.f1.docx]
